# Astrocytes Modify Migration of PBMCs Induced by β-Amyloid in a Blood-Brain Barrier *in vitro* Model

**DOI:** 10.3389/fncel.2019.00337

**Published:** 2019-07-23

**Authors:** Simona Federica Spampinato, Sara Merlo, Evelina Fagone, Mary Fruciano, Cristina Barbagallo, Takashi Kanda, Yasuteru Sano, Michele Purrello, Carlo Vancheri, Marco Ragusa, Maria Angela Sortino

**Affiliations:** ^1^Section of Pharmacology, Department of Biomedical and Biotechnological Sciences, University of Catania, Catania, Italy; ^2^Department of Clinical and Experimental Medicine, University of Catania, Catania, Italy; ^3^Section of Biology and Genetics, Department of Biomedical and Biotechnological Sciences, University of Catania, Catania, Italy; ^4^Department of Neurology and Clinical Neuroscience, Yamaguchi University, Yamaguchi, Japan; ^5^Oasi Research Institute – IRCCS, Troina, Italy

**Keywords:** Alzheimer’s disease, endothelial cells, cytokines, leukocytes, ICAM-1, VE-cadherin, glycosylation

## Abstract

**Background:**

The brain is protected by the blood-brain barrier (BBB), constituted by endothelial cells supported by pericytes and astrocytes. In Alzheimer’s disease a dysregulation of the BBB occurs since the early phases of the disease leading to an increased access of solutes and immune cells that can participate to the central inflammatory response. Here we investigated whether astrocytes may influence endothelial-leukocytes interaction in the presence of amyloid-β (Aβ).

**Methods:**

We used an *in vitro* BBB model, where endothelial cells, cultured alone or with astrocytes were exposed for 5 h to Aβ, both under resting or inflammatory conditions (TNFα and IFNγ), to evaluate endothelial barrier properties, as well as transendothelial migration of peripheral blood mononuclear cells (PBMCs).

**Results:**

In the co-culture model, barrier permeability to solutes was increased by all treatments, but migration was only observed in inflammatory conditions and was prevented by Aβ treatment. On the contrary, in endothelial monocultures, Aβ induced leukocytes migration under resting conditions and did not modify that induced by inflammatory cytokines. In endothelial astrocyte co-cultures, a low molecular weight (MW) isoform of the adhesion molecule ICAM-1, important to allow interaction with PBMCs, was increased after 5 h exposure to inflammatory cytokines, an effect that was prevented by Aβ. This modulation by Aβ was not observed in endothelial monocultures. In addition, endothelial expression of β-1,4-N-acetylglucosaminyltransferase III (Gnt-III), responsible for the formation of the low MW ICAM-1 isoform, was enhanced in inflammatory conditions, but negatively modulated by Aβ only in the co-culture model. miR-200b, increased in astrocytes following Aβ treatment and may represent one of the factors involved in the control of Gnt-III expression.

**Conclusion:**

These data point out that, at least in the early phases of Aβ exposure, astrocytes play a role in the modulation of leukocytes migration through the endothelial layer.

## Introduction

Alzheimer’s disease is a neurodegenerative disorder classically associated with amyloid β peptide (Aβ) plaques and phosphorylated τ tangles. The neuronal degeneration that characterizes the disease is associated with an inflammatory state in the CNS: microgliosis and astrocytosis are often recognized in the brain of AD patients. Growing evidence shows that in neuropathological conditions the CNS can also be infiltrated by peripheral inflammatory cells. In particular, the presence of monocytes and lymphocytes, as well as cytokines production, have been described in the brain of AD patients and in AD animal models (Sugimoto et al., 2003; Zand et al., 2005; Ferrara et al., 2007). This is not the case in physiological conditions, in which the access of immune cells to the CNS is strictly regulated, and only few cells go through the BBB to undertake immune surveillance (Argaw et al., 2012). The BBB is in fact constituted by specialized endothelial cells that, together with astrocytes and pericytes, regulate the access of solutes, drugs and cells of the immune system to the CNS. In AD there are several hints of BBB dysfunction, as indicated by leakage, often observed in patients with early AD and described in post mortem studies (de la Torre, 2002; Jellinger and Attems, 2005; Keller, 2013). Evidence also exists to support the idea that, in AD, BBB changes precede neurodegenerative events (Bell and Zlokovic, 2009; Zhao et al., 2015). In a BBB *in vitro* model, Aβ exposure modifies the distribution of TJ proteins (Marco and Skaper, 2006; Gonzalez-Velasquez et al., 2008; Nagababu et al., 2009) and induces the expression of adhesion molecules ICAM-1 and VCAM-1 (Liu et al., 2011), all hallmarks of BBB dysfunction.

Endothelial cells express adhesion molecules able to capture PBMCs from the blood stream (Kansas, 1996). Once they enter in contact with the endothelial monolayer, PBMCs engage a strict interaction with adhesion molecules, redistribute in perijunctional areas where, resistant to the blood shear stress, can initiate their migration through the endothelial layer (Rahman and Fazal, 2009; Dragoni et al., 2017) in a mechanism that implicates also VE-cadherin mobilization (Muller, 2016b). Once they cross the BBB, leukocytes, interacting with the cells of the CNS (primarily microglia and astrocytes), may be involved in the process of neuroinflammation, further precipitating neuronal death.

Our interest was to evaluate whether astrocytes, known to cooperate with endothelial cells in BBB function, may influence leukocytes migration toward the CNS. Endothelial cells and astrocytes co-cultures represent an *in vitro* model that more closely reproduces BBB features (Abbott et al., 2006). Using human microvascular brain endothelial cells co-cultured with human astrocytic cell line, we have recently demonstrated that astrocytes play an essential role in modulating the response of endothelial cells to Aβ. Through the release of VEGF from astrocytes, Aβ increased the response of endothelial cells, inducing changes in TJ expression and increased BBB permeability (Spampinato et al., 2017). Here we studied the effects of astrocytes in the modulation of PBMCs’ migration through the endothelial layer exposed to Aβ under resting conditions and following inflammatory stimulation, a condition that favors PBMCs migration in the *in vitro* BBB model (Takeshita et al., 2014; Spampinato et al., 2015).

## Materials and Methods

### Reagents

All cell culture plastics were from BD Falcon (Milan, Italy). Polycarbonate membrane transwell inserts (3 and 8 μm pores), collagen I rat tail and lymphocyte separation medium were provided by Corning (Milan, Italy). MCDB-131 medium, RPMI 1640 without phenol red, fetal bovine serum (FBS), and all media supplements, unless otherwise specified, were from Invitrogen SRL (Milan, Italy). EGM-2 SingleQuots^TM^ was provided by Lonza, (Basel, Switzerland). Astrocyte medium was supplemented with astrocyte media kit (both from ScienCell Research Laboratories, Carlsbad, CA). β-amyloid (Aβ)1–42 peptide (Innovagen, Lund, Sweden) was solubilized in dimethyl-sulfoxide as a 5 mM stock solution. Subsequent dilutions were made in medium. A concentrated solution of Aβ(1–42), 100 μM, was aggregated by overnight incubation at 4°C, followed by freeze-thaw cycles for enrichment in oligomers, as previously described. For experiments, Aβ(1–42) was diluted in culture medium to a final concentration of 2.5 μM. The state of oligomerization of the peptide was evaluated with western blot analysis showing a mixture of monomers, dimers, tetramers and different size oligomers (as shown previously) (Merlo and Sortino, 2012), and its aggregation was not modified after incubation in medium either for 5 or 18 h (timepoints used in our experimental protocols, not shown). TNFα and IFNγ were from Peprotech Inc. (London, United Kingdom).

### Cell Culture

TY-10 cells, a brain microvascular endothelial cell line, and astrocytes (hAST), were adult human cells immortalized after transfection with plasmid expressing temperature sensitive Simian virus-40 large T-antigen (ts-SV40- LT) and the catalytic subunit of human telomerase, as previously described (Haruki et al., 2013; Sano et al., 2013). Both cell lines were developed at Yamaguchi University (Japan), in the labs of Dr. Sano and Kanda. TY-10 cells were grown in MCDB-131 media supplemented with EGM-2 SingleQuots^TM^ and 20% heat-inactivated FBS, while hAST were grown in astrocyte media containing 2% heat-inactivated FBS, astrocyte growth supplement and penicillin/streptomycin solution as provided with the Astrocyte media kit. For experiments, both TY-10 cells and hAST were grown at 33°C until confluency was reached and then transferred at 37°C, where they exhibit growth arrest and differentiation; when transferred at 37°C, all cultures were maintained in astrocyte culture medium Treatments, all carried out in astrocyte medium, were performed when transendothelial electrical resistance (TEER) values were stable (34.05 ± 0.5 Ω cm^2^), as previously reported (Sano et al., 2013; Spampinato et al., 2017). When co-culture models were used, both endothelial cells and astrocytes were exposed to treatments simultaneously.

### PBMC Isolation

Peripheral blood mononuclear cells were isolated from fresh heparinized blood of healthy subjects by density centrifugation with Lymphocyte Separation Medium (Corning, Fisher Scientific, Milan, Italy) as previously described (Man et al., 2008). For transmigration assay, PBMCs were suspended in transendothelial migration (TEM) buffer (RPMI 1640 without phenol red, 1% bovine serum albumin, Hepes, L-glutamine, Na-pyruvate, MEM non-essential amino acids).

### FITC-Dextran Permeability Assay

In the co-culture model, hAST (9 × 10^4^ cells) were plated on the bottom of a 24-well multiwell plate; after 1 h endothelial cells (1.2 × 10^5^ cells) were plated on Collagen type-I-coated polycarbonate transwell inserts (3 μm pores), transferred on the same plate where the co-culture was grown in astrocyte medium. After 3 days at 33°C, cells were transferred at 37°C to allow cell differentiation for 2 more days. After treatment, inserts were equilibrated in the “assay medium” (in phenol red-free DMEM medium supplemented with 1% FBS) for 30 min at 37°C. Solute permeability was assessed using 10 kDa FITC-conjugated dextran (1 mg/mL Sigma-Aldrich) that was applied to the luminal compartment. Samples (100 μl) were collected from the abluminal compartment after 30 min. Sample fluorescence was measured at 485/520 nm (excitation/emission) using Varioskan^TM^ LUX multimode microplate reader (Thermo Fisher, Milan, Italy). Fluorescence intensity values were plotted on the *Y*-axis and represented as % of control.

### Migration Assay

For the static transmigration assay, 6.5 mm polycarbonate membrane cell culture inserts with 8.0 μm *pore* (Corning^®^ Transwell^®^) were used. hAST (3 × 10^5^ cells per wells) were seeded on the abluminal side of the membrane, and after attachment, inserts were flipped and TY-10 (5 × 10^5^ per membrane) seeded on the luminal side. Co-cultures were grown in astrocyte medium for 2 days at 33°C, and then kept for 2 days at 37°C before any kind of treatment, as indicated. Cells were activated with TNFα (10 U/ml) and IFNγ (5 U/ml) in astrocyte medium for 5–18 h at 37°C and co-treated with Aβ (2.5 μM). CXCL12 (50 ng/ml in TEM buffer, Peprotech) was applied to the apical endothelial layer and incubated for 15 min at 37°C. FBS 1% was used as chemoattractant in the abluminal side. PBMCs (2.8 × 10^6^ cells per assay) were added on the top of the endothelial layer. The assay was ended after a total of 18 h. Migrated PBMCs were recovered from the bottom chamber and enumerated by a hemocytometer.

### 3-[4,5-Dimethylthiazol-2-yl]-2,5-Diphenyltetrazoliumbromide (MTT) Viability Assay

Endothelial cells were incubated with 1 mg/ml MTT substrate (Sigma-Aldrich) for 2 h at 37°C. Dimethyl sulfoxide was added to obtain cell lysis and solubilization of formazan resulting from MTT reduction by viable cells mitochondrial activity. Absorbance at 545 nm was then measured with a Varioskan^TM^ Flash Multimode Reader.

### Trypan Blue Exclusion Assay

Dead cell staining was examined by Trypan blue (Sigma-Aldrich; 0.4% for 10 min) after 5 h treatments of endothelial cells, cultured alone or in the presence of astrocytes, with either Aβ, T&I or both. Stained endothelial cells, i.e., dead cells, were manually counted from three to five random fields per well with phase contrast microscopy at a 20× magnification. Each experiment was repeated twice, and every treatment/condition was performed in triplicate.

### Western Blot

Cells were harvested with RIPA protein extraction reagent (Sigma-Aldrich) supplemented with protease inhibitors, and protein concentration determined using the Bradford reagent (Sigma-Aldrich). 30 μg of each sample were separated by SDS-PAGE and transferred to nitrocellulose membranes (Hybond ECL, Amersham Biosciences Europe GmbH, Milan, Italy). Membranes were blocked with Odyssey blocking buffer (LI-COR Biotechnology GmbH, Bad Homburg, Germany) diluted 1:1 with PBS for 30 min and probed with the following primary antibodies overnight: rabbit anti-Claudin-5 (1:200, Invitrogen, Cat. #34-1600, Lot. #RB232835), mouse anti-ICAM-1 (1:800, SantaCruz Biotechnologies, Santa Cruz, CA Cat. #sc-8439, Lot. #B2316), rabbit anti-VE-Cadherin (1:1000, Cell signaling, Cat. # 2500, Lot #D87F2), mouse anti-GAPDH (1:800, Millipore, Cat. #MAB374, Lot #2742734), rabbit anti-β-actin (1:1000, Sigma-Aldrich, Cat. #A2066, Lot #095M46V). Membranes were then processed for immunodetection using specific fluorescent IRDye^®^680- or IRDye^®^800-conjugated secondary antibodies (LI-COR, Cat. # 926-32211, Lot #C20906-02 and Cat. #926-68070, Lot #C20925-04). Detection of specific bands was carried out using the LI-COR Odyssey^®^ Infrared Imaging System (LI-COR Bioscience). Band intensity was analyzed using the image processing software “Image J” developed by NIH and in public domain.

### Peptide N-Glycosidase F (PNGase F) Digestion

Fifty μg of proteins, extracted from T&I-exposed endothelial cells, were incubated with 5 units of PNGase F (Sigma-Aldrich) for 3 h according to the manufacturer’s protocol. A parallel experiment was conducted under the same conditions, except that PNGase F was omitted. At the end of the reaction, samples were separated by gel electrophoresis, blotted and detected with the anti-ICAM-1 antibody as previously described.

### Immunocytochemistry

Endothelial cells were plated on collagen I rat tail-coated coverslips. When grown in co-culture, astrocytes plated on the top of *transwell* tissue culture inserts (BD Falcon), were transferred on the endothelial monolayer. Cultures were checked for confluency and then kept for 2 days at 37°C before treatment, as indicated. Endothelial cells were fixed in ice-cold acetone for 15 min and subsequently in ice-cold methanol for 20 min. Anti-VE-cadherin primary antibody (1:100, SantaCruz Biotech, Cat. # sc-52751, Lot #JO914), was incubated in 0.1% Triton X-100 at 4°C overnight. Secondary antibody (Donkey anti-rabbit Alexa Fluor 546, Invitrogen, Cat. # A100040, Lot #1218269) was incubated for 45 min at room temperature. Cells were imaged using an epifluorescent microscope Zeiss Observer.Z1 microscope equipped with the Apotome.2 acquisition system connected to a digital camera.

### Real Time PCR

Total RNA was extracted from cell cultures using the RNeasy plus Mini Kit (Qiagen, Milan). 1 μg of RNA was used for cDNA synthesis, using the Superscript-VILO kit (Invitrogen) according to manufacturer’s instruction. Quantitative real-time PCR was performed with Rotor Gene Q using QuantiNova SYBR Green PCR Kit (Qiagen). The melting curves obtained after each PCR amplification reaction confirmed the specificity of the 2-[N-(3-dimethylaminopropyl)-N-propylamino]-4- [2,3-dihydro-3-methyl-(benzo-1,3-thiazol-2-yl)-methyli-dene]-1-phenyl-quinolinium (SYBR Green assays). The following quantitec primers (Qiagen, Milan, Italy) were used: human ICAM-1 (QT00074900), human MGAT3-1 (QT00216006), using human RPLP0 (QT00075012) as endogenous control. Expression fold changes were calculated by applying the 2-ΔΔCt method.

### Micro RNA

Expression of miR-200b was investigated by single TaqMan assay (Thermo Fisher Scientific), using U6 snRNA as endogenous control. MiRNA-specific cDNA was retrotranscribed from 100 ng of total RNA through TaqMan microRNA Reverse Transcription Kit (Thermo Fisher Scientific) and successively amplified in a 7900HT Fast Real-Time PCR System (Thermo Fisher Scientific), using TaqMan Universal Master Mix II, no UNG (Thermo Fisher Scientific). Expression fold changes were calculated by applying the 2-ΔΔCt method.

### Statistical Analysis

All data are expressed as means ± SEM of 3–6 different experiments each run in duplicates or in triplicates as specified in the figure legends. Data were analyzed by one-way ANOVA followed by Newman–Keuls test for significance. *p* < 0.05 was taken as the criterion for statistical significance when three or more conditions were compared. Student’s *t*-test was applied between two groups. *p* < 0.05 was taken as the criterion for statistical significance.

## Results

**FIGURE 1:**
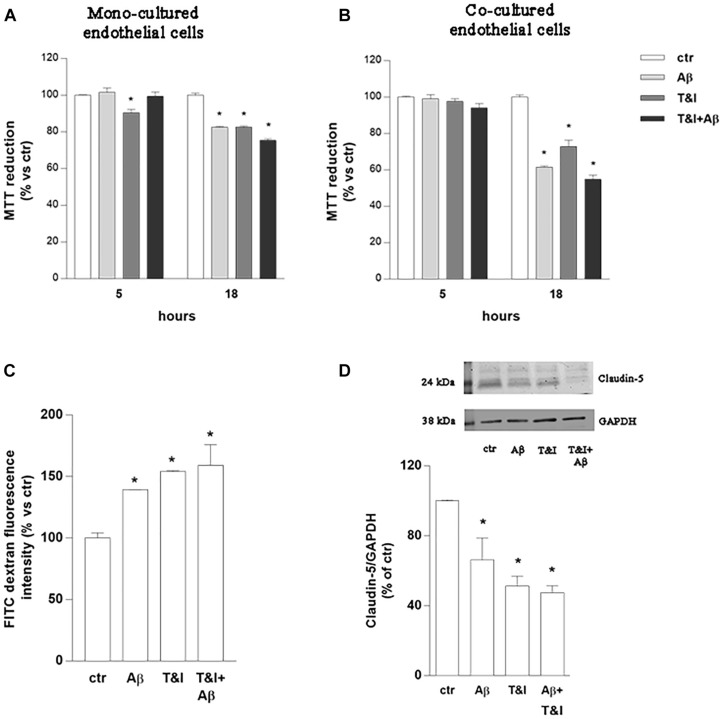
Endothelial permeability is affected by Aβ under resting and inflammatory conditions. Endothelial mono-cultures **(A)** and co-cultures **(B)** were exposed to Aβ 1–42 (Aβ, 2.5 μM), TNFα+IFNγ (10 U/ml and 5 U/ml respectively, T&I) or their combination (Aβ+T&I). MTT assay was used to evaluate endothelial viability after different time points (5 and 18 h). Barrier properties of endothelial-astrocytes co-cultures, exposed for 5 h to Aβ (2.5 μM), T&I (U/ml and 5 U/ml respectively) or their combination (Aβ+T&I), were examined by measuring FITC-conjugated dextran permeability through the monolayer, 30 min after addition of the dye **(C)**, and the expression of Claudin-5 by western blot analysis **(D)**. Data are expressed as percentage of control viability **(A,B)**. Barrier permeability is expressed as percentage of control of FITC-dextran-10 kDa fluorescent intensity, plotted on the *Y*-axis **(C)**. Data are mean ± SEM of 3 independent experiments, each run in duplicate. ^*^p < 0.05 versus control. Significance was assessed by one-way ANOVA followed by Newman–Keuls test.

Endothelial cell cultures were exposed to Aβ (2.5 μM), TNF-α and IFNγ (T&I 10 U/ml and 5 U/ml, respectively) or their association (Aβ+T&I), and viability was evaluated at different time points (5, 18 h) using MTT assay (Figure 1A). Slight but significant endothelial cell death (10%) was already observed after 5 h of T&I exposure. Aβ *per se* induced endothelial cell death only after 18 h treatment, and similar effects were observed also when endothelial cells were exposed to both T&I and Aβ. The same protocol was applied on astrocytic cultures, whose viability was not affected by treatments at the different time points (data not shown). Astrocytes and endothelial cell lines were then co-cultured on inserts of polycarbonate membranes and exposed to treatments for 5 or 18 h. In the co-culture model, in contrast to what observed in the endothelial monolayer alone, T&I did not affect cell viability at 5 h, but endothelial cell death occurred when treatments were prolonged for 18 h (Figure 1B). Endothelial viability was assessed also by trypan blue exclusion assay, confirming that, after 5 h, T&I slightly induced cell death, only in the endothelial mono-cultures (Supplementary Table 1). These results prompted us to consider the 5 h time-point to analyze effects on barrier properties, without occurring in a massive endothelial death. Endothelial barrier properties were then tested by evaluating permeability to FITC-conjugated dextran, an indirect measure of the tightness of TJ; exposure of endothelial-astrocytes co-cultures for 5 h to Aβ, T&I or their combination significantly increased dextran permeability (Figure 1C), an effect supported by the reduced expression of Claudin-5, as indicated by western blot analysis (Figure 1D).

**FIGURE 2:**
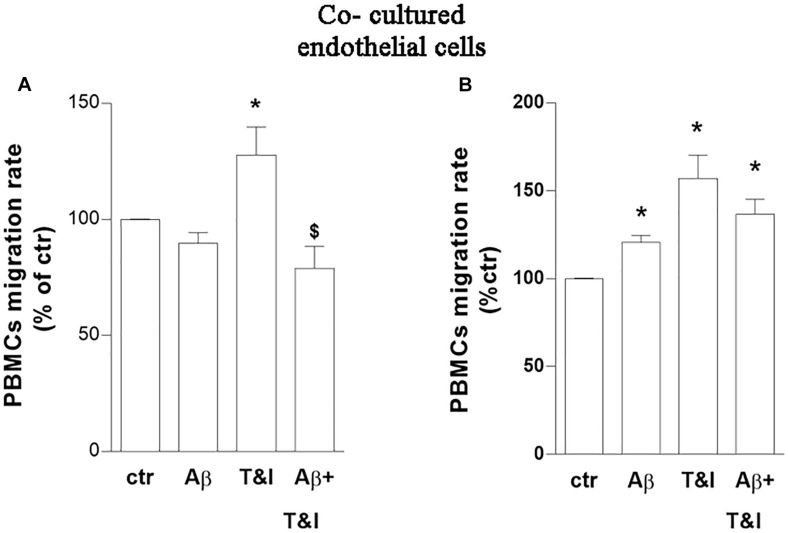
PBMCs migration induced by inflammatory cytokines is prevented by a short-term exposure to Aβ. Endothelial cells co-cultured with astrocytes were exposed to Aβ 1–42 (Aβ, 2.5 μM), TNFα+IFNγ (10 U/ml and 5 U/ml respectively, T&I) or their combination (Aβ+T&I) for 5 **(A)** or 18 h **(B)**. Effects of treatments were examined by evaluating the number of PBMCs migrating through the barrier after 18 h. PBMCs migration represents the ratio of migrated cells through the barrier, given a constant input, expressed as percentage of migration in control conditions. Data are mean ± SEM of 5 **(A)** or 3 **(B)** independent experiments, each run in duplicate. ^*^p < 0.05 versus control, ^$^*p* < 0.05 versus T&I. Significance was assessed by one-way ANOVA followed by Newman–Keuls test.

The capability of PBMCs to migrate through the *in vitro* BBB model, under these experimental conditions, was then evaluated. Co-cultures were exposed to Aβ (2.5 μM), T&I (10 U/ml and 5 U/ml, respectively) and their association for 5 h. PBMCs were plated on top of the endothelial (luminal) side of the inserts, and allowed to transmigrate toward the abluminal side up to 18 h. PBMCs crossed more easily the *in vitro* BBB previously exposed to T&I (Figure 2A and Supplementary Table 2).

Aβ, by itself, only slightly and not significantly modified migration rate of PBMCs in basal conditions, but completely prevented PBMCs’ migration induced by T&I (Figure 2A). A prolonged exposure to treatments (18 h) increased instead the migration rate of PBMCs in all the conditions examined (Figure 2B and Supplementary Table 2). The phenotype of migrated cells was analyzed by flow cytometry. After migration, there was an enrichment of CD3+ cells, and more than 50% of those were CD4+ cells. Migrated PBMCs phenotype was not affected by treatments (not shown) (Supplementary Table 3).

**FIGURE 3:**
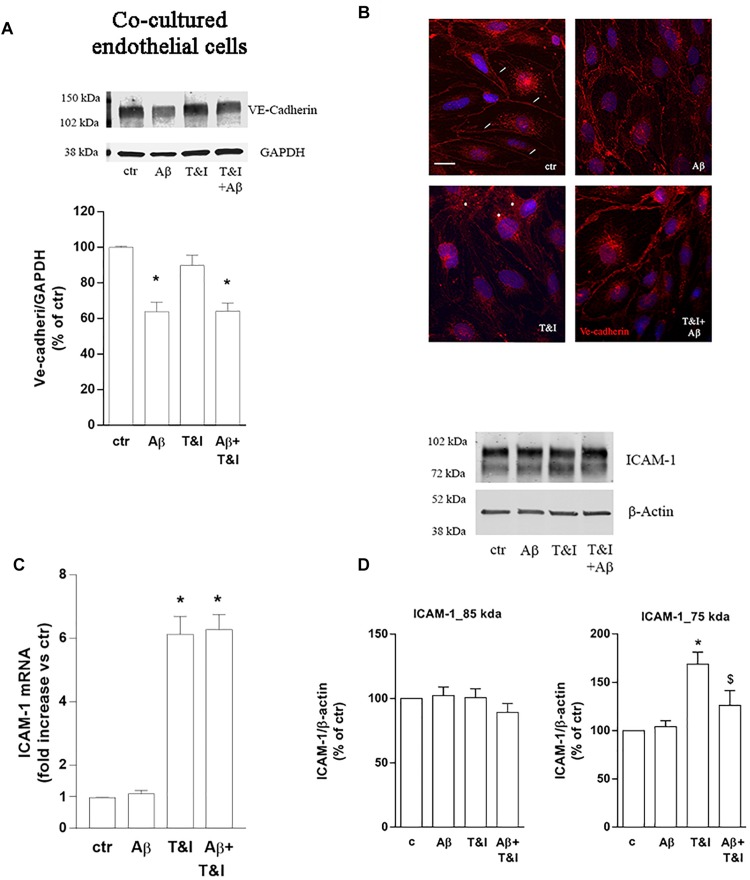
Aβ treatment affects the expression of VE-cadherin and prevents T&I-induced overexpression of hypermannosylated ICAM-1. Endothelial astrocytes co-cultures were exposed for 5 h to Aβ (2.5 μM), T&I (10 U/ml and 5 U/ml respectively) or their combination (Aβ+T&I), and the expression and cellular localization of VE-cadherin were examined by western blot **(A)** and immunofluorescence analysis **(B)**. ICAM-1 mRNA levels were evaluated in endothelial cells co-cultured with astrocytes and exposed to treatments for 5 h **(C)**. Protein expression of ICAM-1 in endothelial cells co-cultured with astrocytes and exposed to treatments was evaluated by western blot analysis. Densitometric analysis of the two isoform bands (85 and 75 kDa) are reported in **(D)**. mRNA levels are reported as fold increase versus control **(C)**. Ve-cadherin is represented in red, while 4′6-diamidino-2-phenylindole (DAPI) is used to counterstain nuclei. Scale bar = 10 μm. Data are mean ± SEM of 4 **(A)** 3 **(C)** or 6 **(D)** independent experiments. ^*^p < 0.05 versus control, ^$^*p* < 0.05 versus T&I. Significance was assessed by one-way ANOVA followed by Newman–Keuls test.

The expression of endothelial VE-cadherin, one of the main factors involved in transendothelial migration, was evaluated by western blot analysis after exposure of the co-cultures to treatments for 5 h. Aβ, both under resting and inflammatory conditions (Aβ+T&I), significantly reduced VE-cadherin expression (Figure 3A). Immunocytochemical analysis revealed that the cell boundaries localization of the junctional protein observed in resting condition (small arrow, Figure 3B) was less defined after treatments and perinuclear staining appeared in particular in T&I-treated endothelial cells (asterisks in Figure 3B). Endothelial expression of the adhesion molecule ICAM-1 was further examined. RT-PCR analysis indicated that, while Aβ *per se* had no effect, both T&I and T&I+Aβ significantly induced ICAM-1 mRNA (Figure 3C). Western blot analysis revealed the presence of two distinct ICAM-1 isoforms: a band that migrated at ∼85 kDa, and a lower one at ∼75 kDa. While the expression of the higher band was not affected by treatments, the 75 kDa band was significantly induced by T&I exposure (Figure 3D). Aβ slightly modified the expression of the lower band in basal conditions, but completely prevented the 75 kDa ICAM-1 overexpression induced by T&I (Figure 3D).

**FIGURE 4:**
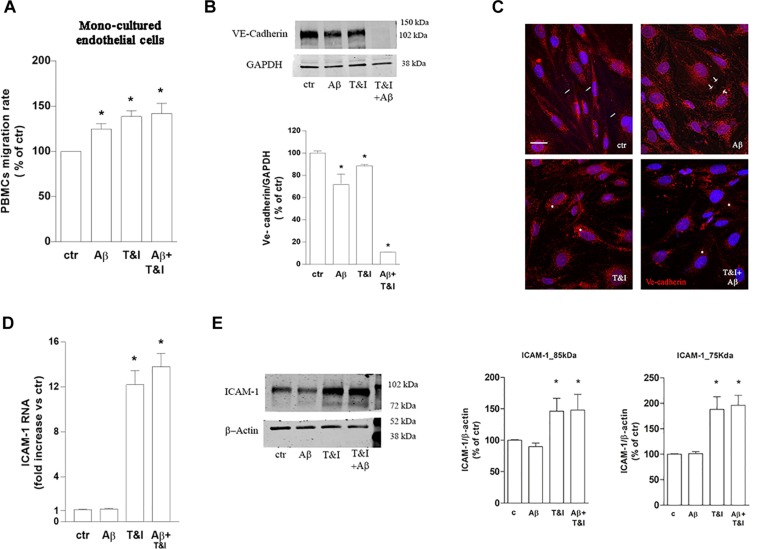
PBMCs migration through endothelial monolayer is induced by Aβ treatments under inflammatory conditions. PBMCs migration through a barrier constituted by endothelial mono-cultures exposed for 5 h to Aβ (2.5 μM), T&I (10 U/ml and 5 U/ml respectively) or their combination (Aβ+T&I), is evaluated after 18 h migration assay **(A)**. Western blot expression **(B)** and immunocytochemical analysis of VE-cadherin **(C)**, as well as and ICAM-1 mRNA levels **(D)** and protein expression **(E)** were evaluated in endothelial mono-cultures exposed to treatments for 5 h. PBMCs migration represents the ratio of migrated cells through the barrier given a constant input after 18 h assay, and then expressed as percentage of migration in control conditions **(A)**. Data are mean ± SEM of 3 (**A**, each run in duplicate, and **C**) or 4 **(B,D)** independent experiments. ^*^p < 0.05 versus control. Significance was assessed by one-way ANOVA followed by Newman–Keuls test.

To assess the role of astrocytes in the observed effects, parallel experiments with the same conditions as described above, were carried out on endothelial monocultures. PBMCs transmigration was induced by 5 h exposure to Aβ (2.5 μM) as well as by T&I (10 U/ml and 5 U/ml, respectively) and by Aβ+T&I (Figure 4A and Supplementary Table 2). VE-cadherin expression was reduced by Aβ, T&I and completely absent when the two treatments were added together (Figure 4B). Although in basal conditions VE-cadherin localization at cell boundaries was less defined if compared to co-cultured endothelial cells (Figure 4C, ctr, small arrows), treatments completely altered its expression and cellular localization. Aβ induced the formation of gaps between cells (Figure 4C, Aβ, arrows) with loss of contact through VE-cadherin, while in the presence of T&I, the protein was internalized in the perinuclear area (Figure 4C, T&I, asterisks). When endothelial monolayer was exposed to T&I+Aβ, total expression of the protein was reduced and its boundaries localization completely prevented (Figure 4C). ICAM-1 mRNA was significantly increased by T&I; Aβ, when given alone, did not modify its expression, nor did it prevent its induction under inflammatory conditions (Aβ+T&I, Figure 4D). Western blot analysis of ICAM-1 expression revealed that the 75 kDa isoform band was only barely visible in resting conditions, while it clearly appeared after endothelial exposure to T&I, and this effect was not prevented by Aβ co-treatment (Figure 4E). The expression of the 85 kDa isoform was instead clearly visible in all conditions, and its levels were increased after 5 h T&I exposure as well as after Aβ+T&I (Figure 4E).

**FIGURE 5:**
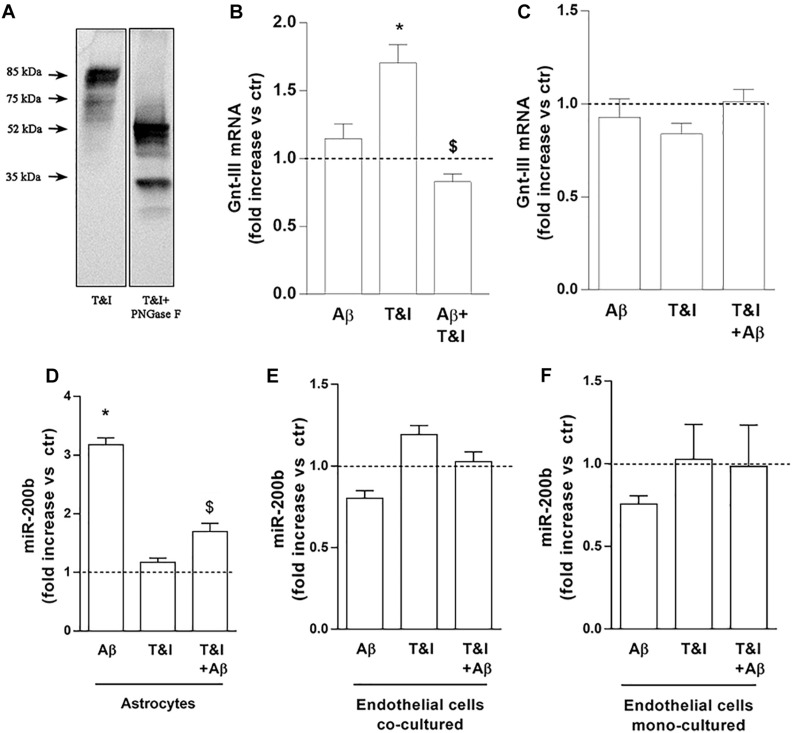
Endothelial expression of Gnt-III is differently modulated in the presence of astrocytes. The expression of ICAM-1 after 5 U PNGase enzymatic digestion was evaluated by electrophoretic separation of lysates derived from 5 h T&I-exposed endothelial co-cultured cells **(A)**. mRNA levels of Gnt-III were evaluated in endothelial cells both co-cultured with astrocytes **(B)** or in mono-cultures **(C)** and exposed for 5 h to Aβ (2.5 μM), T&I (10 and 5 U/ml respectively) or their combination (Aβ+T&I). The expression of miR-200b was examined in astrocytes co-cultured with endothelial cells **(D)**, endothelial cells co-cultured with astrocytes **(E)**, or endothelial mono-cultures **(F)**, all exposed to treatments for 5 h. Values are expressed as fold increase versus ctr. Data are mean ± SEM of 3 independent experiments. ^*^p < 0.05 versus control, ^$^*p* < 0.05 versus T&I. Significance was assessed by one-way ANOVA followed by Newman–Keuls test.

The lower ICAM-1 isoform, may represent the product of a post-translational modification, such as N-glycosylation, largely described for ICAM-1 (Scott et al., 2013). To prove that the band we identified was the product of an N-glycosylation, we treated lysates of T&I-exposed endothelial cells, grown in the presence of astrocytes, with 5 U PNGase F for 3 h at 37°C. This enzyme removes all types of N-glycans from glycoproteins and accordingly we observed that both the 85 kDa and 75 kDa bands were shifted to lower MW bands, virtually free of N-glycans (Figure 5A). We tested the expression of Gnt-III, one of the enzymes primarily involved in protein N-glycosylation. In endothelial/astrocytes co-cultures, the expression of Gnt-III mRNA was significantly upregulated after 5 h exposure to T&I. Aβ completely prevented this increase in inflammatory conditions, while being not effective *per se* (Figure 5B). On the contrary, in endothelial monocultures, Gnt-III levels were not modified by any treatment (Figure 5C).

The expression of the microRNA miR-200b was evaluated in astrocytes (Figure 5D) and endothelial cells, either co-cultured with astrocytes (Figure 5E) or grown in monoculture (Figure 5F). In astrocytes, 5 h of exposure to Aβ significantly increased the expression of miR-200b both in resting conditions and after T&I exposure (Figure 5D). In endothelial cells, either grown with astrocytes or in monocultures, the expression of miR-200b was not modified by Aβ or T&I (Figures 5E,F).

## Discussion

The CNS is an immune-privileged environment and, in physiological conditions, the access of immune cells is limited to immune surveillance. Pathological states of the CNS may affect BBB properties, thus increasing the access of immune cells and inducing central inflammation. In our study, we evaluated the effects of a short-term exposure to Aβ on barrier properties. This experimental paradigm was chosen to mimic an early exposure to Aβ as it may occur during the initial phases of AD. The BBB can in fact be affected already in the early stages of the disease, and its dysfunction may contribute to increased Aβ load and neuroinflammation (Montagne et al., 2017; Nation et al., 2019). Our data show that Aβ prevents PBMCs transendothelial migration induced by the inflammatory status, an effect that appears strictly dependent on the presence of astrocytes. Accordingly, we have recently shown that astrocytes play a major role in modulating endothelial response to Aβ by increasing barrier permeability (Spampinato et al., 2017).

According to a common assumption transendothelial migration and vascular permeability are two strictly connected processes so that leaky vessels should facilitate leukocyte extravasation. However, although both processes share several common mechanisms, requiring the weakening of the intercellular connections, tight and adherens junction (TJ and AJ, respectively) (Cerutti and Ridley, 2017), they are distinct and do not necessarily occur together (Muller, 2016a). We started by analyzing the expression of the AJ VE-cadherin that is implicated in both events. VE-cadherin was reduced in all the conditions examined, justifying, together with the diminished Claudin-5 expression, the increased permeability induced by Aβ under inflammatory stimulus. This effect, however, could not be correlated with the reduced PBMCs migration we observed. This is not surprising considering that, although VE-cadherin is involved in both events, its different phosphorylation state may control its function and determine whether vascular permeability or diapedesis occur (Sidibe and Imhof, 2014; Wessel et al., 2014). In the passage through the BBB, besides VE-cadherin and PECAM-1, adhesion molecules such as ICAM-1, are indeed essential for both paracellular and transcellular migration (Lyck et al., 2003; Yang et al., 2005).

ICAM-1 is a cell surface glycoprotein, with a variable MW (75–114 kDa) depending upon extent of tissue-specific glycosylation (oligosaccharides bound either to an oxygen atom, O-glycan, or to a nitrogen atom, N-glycans) (Rahman and Fazal, 2009; Scott and Patel, 2013), that may vary according to the inflammation state (Garcia-Vallejo et al., 2006; Scott et al., 2013). With the support of PNGase F, capable of enzymatically remove all N-glycosylated chains from proteins (Chen C. et al., 2012), we proved that the 85 and 75 kDa bands of ICAM-1, here described, are two differently N-glycosylated forms of the protein. According to type and position of attached sugars and branching, N-glycosylated proteins can be distinguished in complex, hybrid, and high-mannose types (Palmigiano et al., 2018). High mannose glycans seem to be critical for the response of innate immune system (van Kooyk and Rabinovich, 2008). In particular, accumulation of a high mannose ICAM-1 glycoform, with a predicted MW of 75 kDa, is involved in increased monocytes firm-adhesion (Chacko et al., 2011; Scott et al., 2012).

Accordingly, in our model, treatment with T&I in endothelial cells and in endothelial-astrocyte co-cultures increased the expression of the 75 kDa ICAM-1 isoform and induced PBMCs migration. On the contrary, Aβ, at least in the co-culture model, contrasted the T&I-induced ICAM-1 glycoform and reduced PBMCs migration. This effect was not observed in endothelial monocultures, endorsing the hypothesis that astrocytes modulate endothelial response to Aβ under inflammatory conditions. Astrocytes indeed, in the context of the BBB, may influence endothelial barrier function through the release of soluble mediators (Abbott et al., 2006; Alvarez et al., 2013; Spampinato et al., 2015, 2017). In addition, under inflammatory conditions they do overexpress chemokines. In particular, the chemokine CCL2 and IL-8 are known to modulate the expression of ICAM-1 mRNA (Kawai et al., 2009; Li and Nord, 2009). However, in our model, they appeared not to be involved in the modulation of ICAM-1, as suggested by our preliminary data (not shown).

Therefore, we hypothesized that ICAM-1 post-translational process was instead modified. Human MGAT3 gene encodes for Gnt-III, one of the enzymes involved in the biosynthesis of high mannose glycans (Narasimhan et al., 1988; Nishikawa et al., 1992; Taniguchi et al., 1999). The expression of Gnt-III is regulated in a complex way, often in a cell-type specific manner that may involve the MAPK/ERK pathway (Chen C.L. et al., 2012) or microRNAs. We focused our attention on miR-200b that is known to target MGAT3, by binding to its 3′UTR and reducing its expression (Kurcon et al., 2015). Interestingly, both in *in vitro* and *in vivo* models of diabetes, the overexpression of endothelial adhesion molecules (ICAM-1, VCAM-1, Selectins), and of the enzymes implicated in their O-GlcNAcylation induced by high glucose levels, were significantly decreased by miR-200a and miR-200b mimics (Lo et al., 2018). In line with this observation, in astrocytes challenged with inflammatory cytokines, Aβ increased miR-200b expression, supporting a direct correlation with the observed reduction of endothelial Gnt-III levels. This may result in decreased expression of the highly mannosylated ICAM-1 glycoform and ensuing reduction of PBMCs migration. An even greater induction of miR-200b by Aβ was observed in basal conditions, but this effect did not modify Gnt-III expression, revealing that only increased expression of Gnt-III, as occurs in inflammatory conditions, makes its modulation by miR-200b detectable. Astrocytes have already been shown to affect BBB properties through the modulation of endothelial microRNAs (Reijerkerk et al., 2013). In our experimental conditions, changes in miR-200b were not detectable in endothelial cells, either cultured alone or in the presence of astrocytes, suggesting that endothelial cells are only recipient of a modulatory molecule/s released by astrocytes. We cannot exclude that once the miRNA binds to its targets in endothelial cells, it is quickly turned over, and thus no detectable any more, as it has been described in neurons (de la Mata et al., 2015). Several factors or miRNAs may modulate both the expression and activity of the enzyme Gnt-III and should be further investigated in the future.

The decreased PBMCs migration that we describe here, early after Aβ exposure, appears in contrast with data reported in literature indicating that immune cells adhesion and migration across the BBB is facilitated (Theriault et al., 2015), as observed in AD animal models (Zenaro et al., 2015) and in post mortem AD human brains (Togo et al., 2002). Indeed, also in our model, a longer treatment with Aβ (18 h), caused increased PBMC migration The same effect was observed also in endothelial cells cultured in the absence of astrocytes after a short-time treatment, as already reported (Giri et al., 2000, 2002). The *in vitro* model we used here may carry several limitations and very hardly can completely represent the events occurring in a chronic condition such as AD, where BBB properties are the results of the interaction of endothelial cells with astrocytes, pericytes, microglia and neurons. However, this system allows us to better evaluate the effects exerted by Aβ or inflammatory stimuli on each single cell type (i.e., astrocytes or endothelial cells). By dissecting these effects, we could point out that endothelial response was strongly dependent on the presence of astrocytes in the system.

## Conclusion

Our data point out that astrocytes may profoundly affect endothelial response to a noxious stimulus, and that, at least in the early phases of Aβ exposure, they can contribute to operate a kind of compensatory mechanism that has been reported to occur in the early phases of AD (Merlo et al., 2018, 2019). However, whether this reduced PBMCs migration toward the CNS represents a compensatory and protective mechanism is still unknown. While in fact increased access of PBMCs can participate to neuroinflammation, accelerating neuronal degeneration (Yang et al., 2013), on the other side, Aβ plaque load could be constrained due to monocytes phagocytic abilities (Hawkes and McLaurin, 2009; Mildner et al., 2011; Lai and McLaurin, 2012; Malm et al., 2012). Further studies are needed to evaluate the real impact of the reduced PBMCs access as observed in our BBB model. Our data also suggest that the identification of astrocytic mediators affecting endothelial-PBMCs interaction could be important to predict potential therapeutic interventions able to modulate PBMC infiltration in the CNS during pathological conditions.

## Data Availability

The datasets generated for this study are available on request to the corresponding author.

## Author Contributions

SS, MP, CV, and MS conceived the study. MS acquired the funding. SS, SM, EF, MF, CB, and MR investigated the study. TK and YS provided the resources. SS drafted the manuscript. SM and MS reviewed and edited the manuscript.

## Conflict of Interest Statement

The authors declare that the research was conducted in the absence of any commercial or financial relationships that could be construed as a potential conflict of interest.

## Abbreviations

Aβ: β-amyloid
AD: Alzheimer’s disease
AJ: adherens junctions
BBB: blood-brain barrier
CCL2: C-C motif chemokine ligand 2
CNS: central nervous system
Gnt-III: β -1,4-mannosyl-glycoprotein-4- β-N -acetylglucosaminyltransferase
ICAM-1: intercellular adhesion molecule 1
IFN γ: interferon γ
IL-8: interleukin 8
miR: microRNA
MW: molecular weight
PBMCs: peripheral blood mononuclear cells
PECAM-1: platelet endothelial cell adhesion molecule-1
T&I: TNF α + IFN γ
TEM: *trans-endothelial* migration
TJ: tight junctions
TNF α: tumor necrosis factor α
VCAM-1: vascular cell adhesion molecule 1
VE-cadherin: vascular endothelial cadherin.
