# Supplementary material for: Astrocytes Modify Migration of PBMCs Induced by β-Amyloid in a Blood-Brain Barrier in vitro Model
Source: Front Cell Neurosci. 2019 Jul 23;13:337. doi: 10.3389/fncel.2019.00337 (PMC6664149; doi:10.3389/fncel.2019.00337)
Supplement: Supplementary file 1 [file Table_1.DOCX]

Table 1. Number of trypan blue positive cells in endothelial cells either cultured alone or in the presence of astrocytes, and exposed for 5 h to Aβ ( 2.5μM), T&I (10 U/ml and 5 U/ml respectively) or T&I+Aβ

|  | **ctr** | **Aβ** | **T&I** | **T&I+Aβ** |
| --- | --- | --- | --- | --- |
| **EC** | 49,6±2,1 | 50,1±1,5 | 60,7±2,5 | 55,4±1,8 |
| **EC/AC** | 33,1±1,7 | 29,8 ±1,4 | 38,7± 2,1 | 34,4 ±1,7 |

Table 2. Number of migrated PBMCs

a.

|  |  | **ctr** | **Aβ** | **T&I** | **T&I+Aβ** |
| --- | --- | --- | --- | --- | --- |
| **EC** | **5 h** | 210938±21833 | 224063±22234 | 254375±3248 | 224063±10973 |
| **EC/** | **5 h** | 212517±35957 | 188780 ±16576 | 238640± 40011 | 147880 ±30464 |
| **AC** | **18 h** | 227300±54820 | 281389±74593 | 336244±77183 | 333700±85660 |

b.

|  |  | **ctr** | **Aβ** | **T&I** | **T&I+Aβ** |
| --- | --- | --- | --- | --- | --- |
| **EC** | **5 h** | 6,63±0,47 | 7,03±1,49 | 9,08±0,2 | 7,83±0,47 |
| **EC/** | **5 h** | 7,59±1,29 | 6,04 ±0,85 | 8,52± 1,43 | 5,28 ±1,09 |
| **AC** | **18 h** | 16,16±7,44 | 18,61±7,51 | 24,79±12,32 | 22,7±9,51 |

Endothelial monolayer (EC) or endothelial astrocytes co-cultures (EC/AC) were exposed to Aβ (2.5 µM, Aβ), TNF-α and IFNγ (T&I 10 U/ml and 5 U/ml, T&I) or their association (T&I+Aβ) for either 5h or 18h. Transmigration assay was then performed. PBMCs migrated through the in vitro barrier were recovered from the bottom chamber and enumerated by a hemocytometer.

Mean ± SEM of the raw number (a) and the % (b) of applied PBMCs capable of crossing the endothelial layer is reported.

Table 3. Phenotype of migrated PBMCs

|  | **% CD3+** | **% CD4+ cells** |
| --- | --- | --- |
|  | **cells** | **over CD3+** |
| **input** | 46,41±8,53 | 60,62±7,11 |
| **m_ctr** | 67,79±3,18 | 57,06±7,87 |
| **m_Aβ** | 68,74±3,93 | 67,27±6,53 |
| **m_T&I** | 69,13±3,62 | 54,53±3,02 |
| **m_T&I+Aβ** | 68,54±4,75 | 56,67±3,58 |

PBMCs from healthy donors were stained with mouse anti-CD3(1:100, Immunotech) and PE-conjugated anti-CD4 (1:50, Immunotech) and the percentage of positive cells was evaluated using the Amnis® imaging flow cytometer (Millipore). Analysis was performed on PBMCs recovered after their migration through endothelial/ astrocytes co-cultures in basal conditions (m_ctr), and after exposure to Aβ (2.5 µM, m_ Aβ), TNF-α and IFNγ (T&I 10 U/ml and 5 U/ml, m_T&I) or their association (m_ T&I+Aβ). Phenotype of PBMCs not subjected to migration (input), was further analyzed. Mean± SEM of the percentage of anti-CD3+ cells over total PBMCs and of anti-CD4+ cells over CD3+ cells
